# Disseminated Mycobacterium abscessus Mimicking Sarcoidosis: A Case of Successful Outcomes Against Clinical Expectations

**DOI:** 10.7759/cureus.107149

**Published:** 2026-04-16

**Authors:** Rene A Flores Franco, Nicolas J Saab Santiago, Ernesto Ramos-Martínez

**Affiliations:** 1 Internal Medicine, Instituto Mexicano del Seguro Social (IMSS) Regional General Hospital, Chihuahua, MEX; 2 Ophthalmology, Hospital General “Lázaro Cárdenas”, Instituto de Seguridad y Servicios Sociales de los Trabajadores del Estado (ISSSTE), Chihuahua, MEX; 3 Pathology and Laboratory Medicine, Patología e Inmunohistoquímica de Chihuahua, Chihuahua, MEX

**Keywords:** chronic granulomatous disease, chronic uveitis, lupus pernio, multiorgan sarcoidosis, mycobacterium abscessus (m. abscessus)

## Abstract

*Mycobacterium (M.) abscessus* infections are challenging to treat because there are no drug combinations with proven efficacy. As with other mycobacterioses, disseminated infection caused by *M. abscessus* in immunocompetent individuals can be mistaken for other granulomatous diseases, specifically sarcoidosis. Both diseases can affect virtually any organ and share many clinical manifestations. We present the case of a woman with a confirmed *M. abscessus* disseminated infection, but whose progression exhibited clinical characteristics and a response to treatment very similar to that of sarcoidosis. However, the cutaneous and ocular manifestations were a therapeutic challenge considering that the patient presented an intolerance and adverse events to antimicrobials and a partial refractoriness to systemic corticosteroids. A possible link between these two diseases is discussed, and it is recommended that in cases of disseminated *M. abscessus* infections in immunocompetent individuals, with treatment failure, the possibility of sarcoidosis-like behavior must be reconsidered.

## Introduction

*Mycobacterium (M.) abscessus* is an environmental opportunistic microorganism that commonly causes serious infections in immunocompromised individuals and, occasionally, in immunocompetent individuals [1]. Sporadic skin and soft tissue infections caused by *M. abscessus* have been reported following trauma, surgery, injections, catheter insertion, or exposure to contaminated water from ponds or swimming pools [2]. *M. abscessus* infections are difficult to treat because of multidrug resistance, long-term treatment, and adverse events. Even with prolonged antibiotic regimens containing macrolides, treatment response may be unsatisfactory in 24%-56% of cases [3].

Sarcoidosis is a chronic granulomatous disease that can affect virtually any organ, most commonly the skin, lungs, lymph nodes, and eyes. Diagnosing sarcoidosis is challenging due to the lack of specific diagnostic criteria and the variability in its clinical manifestations, which can mimic many other conditions [4]. Numerous case reports support the co-occurrence of sarcoidosis with *M. tuberculosis* infection, particularly in patients from regions with high TB incidence [5]. More rarely, other mycobacteria, such as *M. avium* subspecies, have also been associated with sarcoidosis. The overlap between mycobacteriosis and sarcoidosis can lead to considerable diagnostic ambiguity, especially because both diseases share many clinical manifestations.

Here, we present an illustrative case of how a granulomatous disease initially diagnosed as atypical mycobacteriosis refractory to treatment partially responded to immunosuppressive therapy for sarcoidosis.

## Case presentation

**Figure 1:**
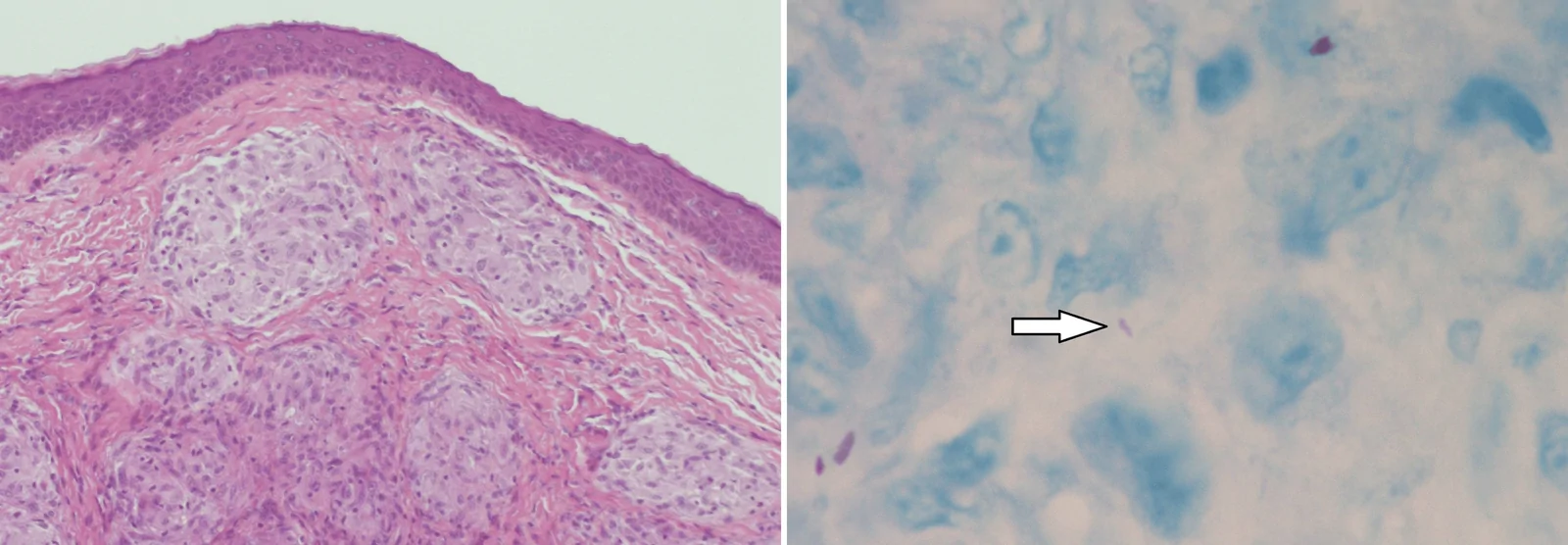
Histopathological findings Left: Histopathology of a hematoxylin-eosin-stained sample of a nasal nodule showing skin with chronic noncaseating granulomatous inflammation affecting both the superficial and reticular dermis. Right: Histopathological sample from an elbow lymph node where some bacilli are evident in the Ziehl-Neelsen stain (arrow).

A 53-year-old woman presented with a three-month history of slowly progressive, erythematous, and painful cutaneous nodules on the nose, accompanied by weight loss, fatigue, fever, and night sweats. She had undergone cosmetic eyelid surgery eight months before presentation. Biopsy of the nasal nodules revealed chronic granulomatous inflammation without mycobacteria on Ziehl-Neelsen staining; however, polymerase chain reaction (PCR) was positive for *M. abscessus* DNA (Figure 1, left). The patient had no concomitant illnesses, and an enzyme-linked immunosorbent assay test for HIV was negative. Initially, she was treated with the following antimicrobial regimen: azithromycin 500 mg daily, rifampin 300 mg every 12 h, minocycline 100 mg every 12 h, and levofloxacin 500 mg daily indefinitely. However, not only did her condition not improve over the following six months, but she also developed gastric intolerance to medications, even more significant weight loss, along with lower limb edema and some lymphadenopathy in the extremities. In addition, her vision began to blur. At this time, a second biopsy taken from a lymph node in her right elbow documented persistent granulomatous inflammation and the presence of a few bacilli on Ziehl-Neelsen staining (Figure 1, right). Periodic acid-Schiff staining did not identify any fungi. By then, a chest X-ray indicated significant mediastinal lymphadenopathy and some interstitial pulmonary changes. Ophthalmological evaluation revealed mutton-fat keratic precipitates in the right eye and yellowish-white vitreous opacities in both eyes, consistent with uveitis.

**Figure 2:**
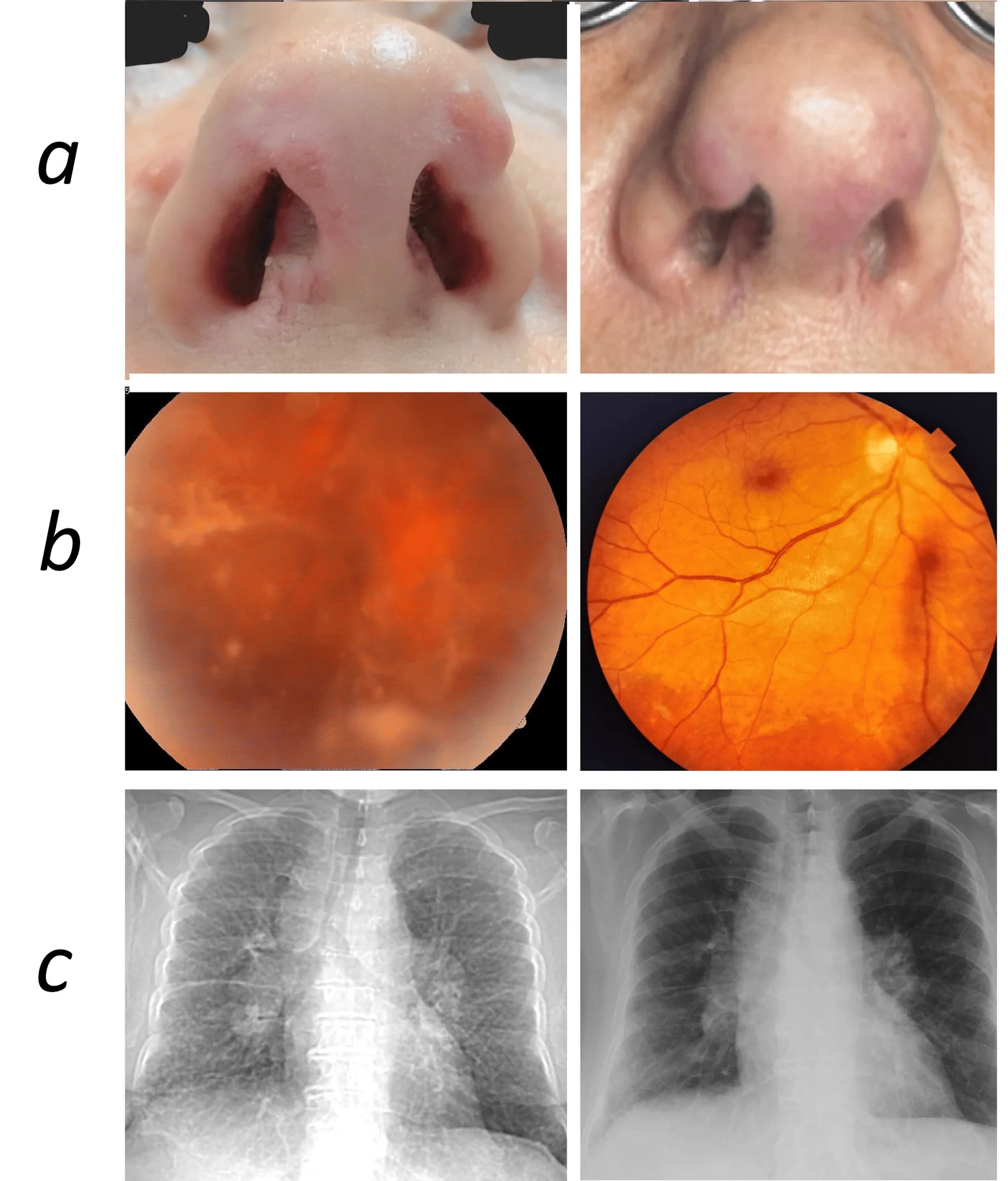
Clinical progress before (left) and one month after (right) systemic corticosteroid therapy a. Nasal nodules. b. Fundoscopy of the left eye showing the resolution of the yellowish-white vitreous opacities suspended. c. Chest X-ray demonstrating a significant decrease in hilar lymphadenopathy.

**Figure 3:**
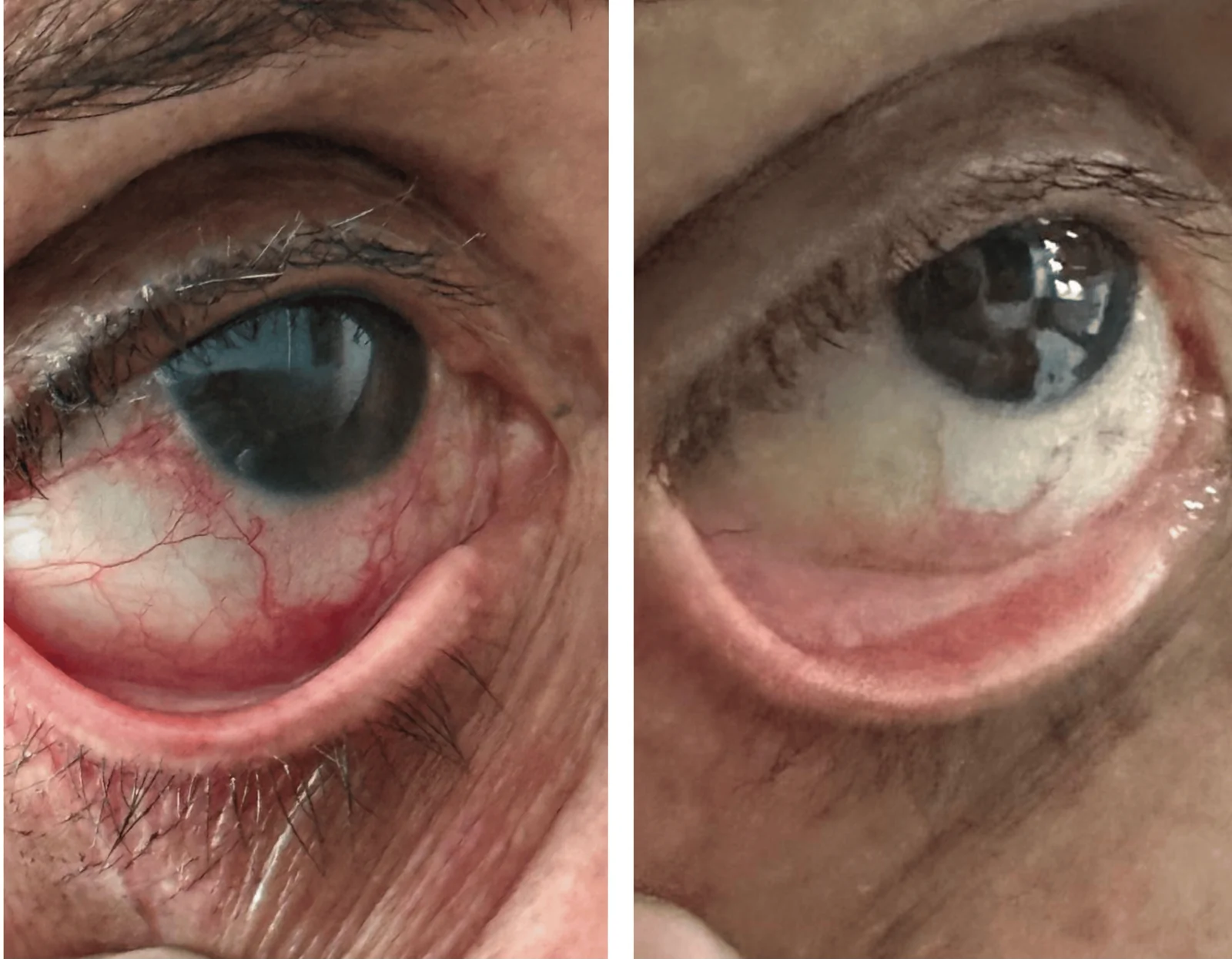
Conjunctival injection before (left) and seven days after (right) deflazacort administration

She remained symptomatic for more than two years from the onset of her illness, and by then, some physicians had already established a poor prognosis. A probable diagnosis of sarcoidosis was considered because of the following: a negative mycobacterial culture and negative PCR for mycobacterial DNA from a new lymphatic tissue sample obtained from the same elbow, a negative QuantiFERON-TB test, and several weeks having elapsed since antibiotic discontinuation. The patient was started on prednisone (50 mg daily). Symptoms improved within a few days, and partial regression of lymphadenopathy was observed. Therefore, prednisone was replaced with methotrexate (12.5 mg once weekly) in the following weeks (Figure 2). However, the nasal lesions persisted, so a new biopsy was performed, which revealed granulomas with caseous necrosis and some isolated mycobacteria on Ziehl-Neelsen staining. Consequently, methotrexate was discontinued. The patient declined another course of antimicrobials and remained under observation for the following weeks. During this period, her ocular symptoms recurred, primarily blurred vision and conjunctival hyperemia. A short course of deflazacort 30 mg twice a day reversed the conjunctival changes, and she did not report a significant deterioration in her visual acuity (Figure 3). Currently, her follow-up has been characterized by remission and exacerbation episodes, especially of nasal and ocular symptoms, but without a deterioration in her quality of life.

## Discussion

This case illustrates how disseminated mycobacteriosis could precipitate a sarcoidosis-like condition. Similar to the course of sarcoidosis, infection by* M. abscessus* is characterized by a tendency toward chronicity, with episodes of flare-ups, an insufficient response to current treatment regimens, and the need for prolonged patient follow-up. In our patient, although the presence of *M. abscessus* was not confirmed by culture, PCR detection and histologic evidence using special stains were considered sufficient to establish the diagnosis. However, the strongest evidence of mycobacterial pathogenicity in sarcoidosis would be the culture of viable organisms from affected tissues since the PCR test cannot distinguish between active infection, latent mycobacteriosis, or residual DNA from previously treated or self-resolving infections [5].

The source of infection in the patient under consideration was not identified, but it may have been related to the previous eyelid surgery. The persistence of symptoms initially led us to consider the possibility of antimicrobial resistance. However, the failure to document the presence of mycobacteria during bacteriological follow-up in new lymphoid tissue samples made it reasonable to reconsider the diagnosis and raise the possibility of a progression of the inflammatory response from a “tuberculous phase” to a “sarcoid phase”. The robust response to immunosuppressive treatment supported this possibility. We began with exploratory doses of systemic corticosteroids and, following clinical improvement, decided to replace them with methotrexate at the doses recommended for treating sarcoidosis [4]. Nevertheless, the nasal nodules did not improve significantly with the treatment (Figure 2a). Lupus pernio (Besnier-Tenneson syndrome) is a cutaneous manifestation of sarcoidosis that presents as smooth, shiny, bluish-red to violaceous nodules and plaques on the head and neck, predominantly on the nose, ears, lips, and cheeks. It rarely resolves spontaneously, is recalcitrant, and may cause cosmetic disfigurement. Its histopathological findings are similar to those produced by sarcoidosis in other affected organs. However, the hallmark histological feature is a noncaseating granuloma with a scant lymphocytic component, known as a naked granuloma. Unlike other forms of cutaneous sarcoidosis, lupus pernio is often accompanied by intrathoracic abnormalities on chest X-rays. Treatment can be difficult and complicated due to its unpredictable course [6,7].

In our patient, the most concerning clinical manifestation was ocular involvement because a delay in initiating corticosteroid treatment could result in irreversible retinal atrophy. The risk of photoreceptor or retinal pigment epithelium atrophy suggests that all intraretinal macular sarcoid lesions should be treated with corticosteroids and/or steroid-sparing agents as soon as possible, ideally before the involvement of the ellipsoid zone or the retinal pigment epithelium [8].

Although no similar cases specifically involving *M. abscessus* as a precipitating agent of sarcoidosis have been reported, a case of a concomitant infection with sarcoidosis has been documented, where the authors suggested that a possible link between the two should not be ignored [9]. Thus, infection and invasion by *M. abscessus* could trigger an abnormal immune response through some mechanism, subsequently promoting granuloma formation. The transition from a mycobacterial-induced granuloma to sarcoidosis involves a shift from a protective, pathogen-clearing immune response to a chronic, self-sustaining, and non-caseating (non-necrotic) inflammatory state in genetically susceptible individuals [10]. The presence of chronic immune stimulation due to persistent microbial antigens has been reported to reduce T-cell function, such as reduced cytokine expression and proliferative capacity, as well as up-regulation of the inhibitory receptor, programmed death-1 (PD-1), all immunologic phenomena associated with elevated antigenic burdens. This T-cell exhaustion results in a reduction in the cell’s ability to optimally respond to its receptor and limit the robustness of the adaptive immune response to foreign antigens [10].

Lastly, in other disseminated mycobacteriosis cases where a sarcoidosis-like behavior has also been observed, the response to antimicrobials has not been entirely satisfactory, and interestingly, treatment with systemic corticosteroids has already been suggested [11].

## Conclusions

Given the high rate of treatment failures in infections caused by *M. abscessus*, our experience suggests that clinicians need to rule out a sarcoidosis-like condition in immunocompetent individuals with disseminated infection, as this may partially alter the patient’s prognosis.
